# Supplementary material for: Inflammatory factors and risk of lung adenocarcinoma: a Mendelian randomization study mediated by blood metabolites
Source: Front Endocrinol (Lausanne). 2024 Aug 27;15:1446863. doi: 10.3389/fendo.2024.1446863 (PMC11384989; doi:10.3389/fendo.2024.1446863)
Supplement: Supplementary file 1 [file DataSheet1.zip › supplementary files/S2/GCST90200725/forest.pdf]

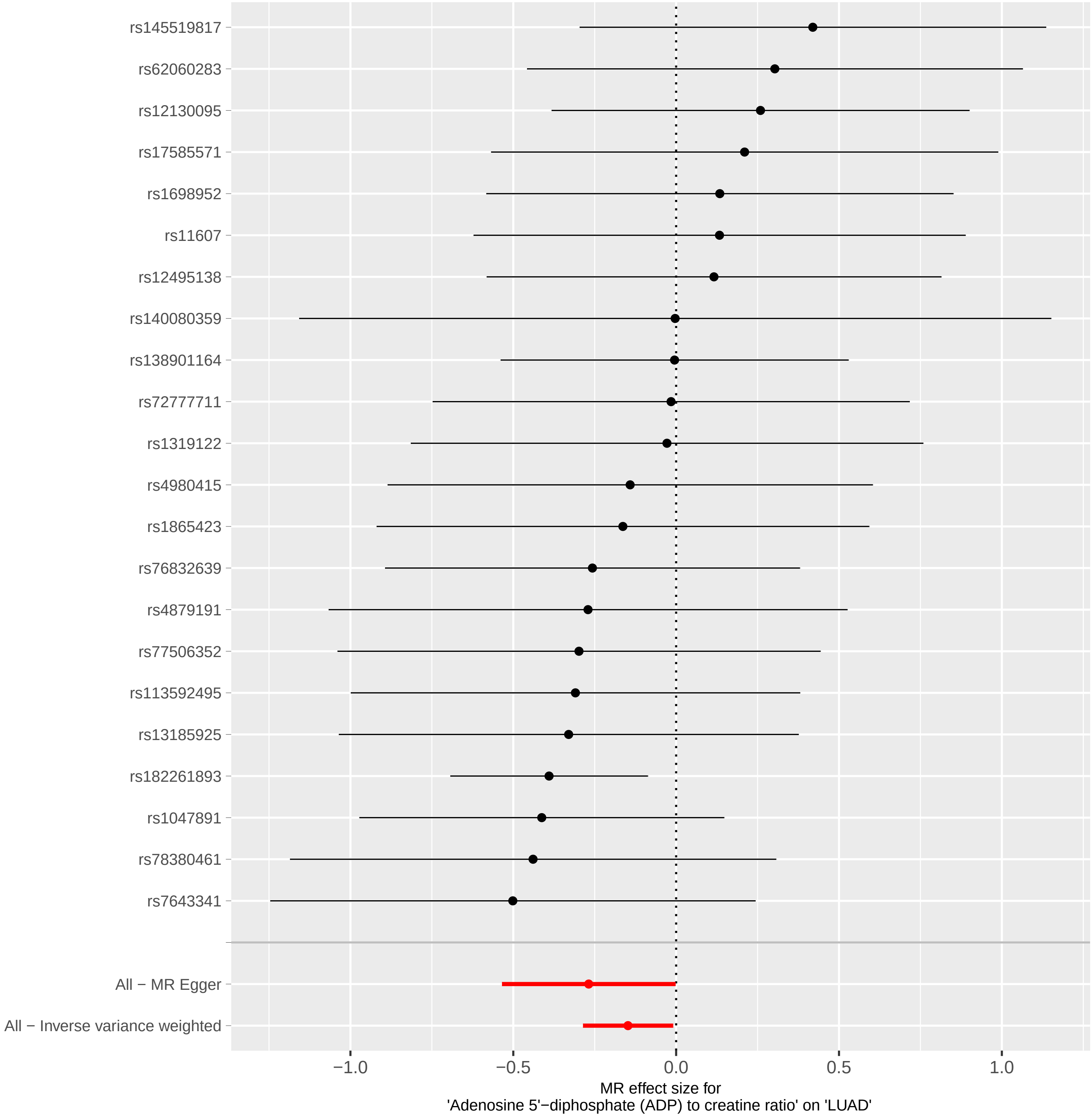
rs145519817
rs62060283
rs12130095
rs17585571
rs1698952
rs11607
rs12495138
rs140080359
rs138901164
rs72777711
rs1319122
rs4980415
rs1865423
rs76832639
rs4879191
rs77506352
rs113592495
rs13185925
rs182261893
rs1047891
rs78380461
rs7643341
All − MR Egger
All − Inverse variance weighted
−1.0
−0.5
0.0
0.5
1.0
MR effect size for
'Adenosine 5'−diphosphate (ADP) to creatine ratio' on 'LUAD'
